# Looming sensitive cortical regions without V1 input: evidence from a patient with bilateral cortical blindness

**DOI:** 10.3389/fnint.2015.00051

**Published:** 2015-10-22

**Authors:** Alexis Hervais-Adelman, Lore B. Legrand, Minye Zhan, Marco Tamietto, Beatrice de Gelder, Alan J. Pegna

**Affiliations:** ^1^Laboratory of Experimental Neuropsychology, Neurology Clinic, Department of Clinical Neuroscience, University of GenevaGeneva, Switzerland; ^2^Brain and Language Lab, Department of Clinical Neuroscience, University of GenevaGeneva, Switzerland; ^3^Faculty of Psychology and Educational Sciences, University of GenevaGeneva, Switzerland; ^4^Department of Cognitive Neuroscience, Faculty of Psychology and Neuroscience, Maastricht UniversityMaastricht, Netherlands; ^5^Department of Psychology, University of TorinoTorino, Italy; ^6^Cognitive and Affective Neuroscience Laboratory, Center of Research on Psychology in Somatic Diseases, Tilburg UniversityTilburg, Netherlands; ^7^Department of Experimental Psychology, University of OxfordOxford, UK; ^8^School of Psychology, University of QueenslandBrisbane, QLD, Australia

**Keywords:** blindsight, motion, looming, fMRI, cortical blindness, hemianopia, MT/V5

## Abstract

Fast and automatic behavioral responses are required to avoid collision with an approaching stimulus. Accordingly, looming stimuli have been found to be highly salient and efficient attractors of attention due to the implication of potential collision and potential threat. Here, we address the question of whether looming motion is processed in the absence of any functional primary visual cortex and consequently without awareness. For this, we investigated a patient (TN) suffering from complete, bilateral damage to his primary visual cortex. Using an fMRI paradigm, we measured TN's brain activation during the presentation of looming, receding, rotating, and static point lights, of which he was unaware. When contrasted with other conditions, looming was found to produce bilateral activation of the middle temporal areas, as well as the superior temporal sulcus and inferior parietal lobe (IPL). The latter are generally thought to be involved in multisensory processing of motion in extrapersonal space, as well as attentional capture and saliency. No activity was found close to the lesioned V1 area. This demonstrates that looming motion is processed in the absence of awareness through direct subcortical projections to areas involved in multisensory processing of motion and saliency that bypass V1.

## Introduction

Moving stimuli are thought to be highly salient and several studies have suggested that motion is a powerful attractor of attention. For example, in a visual search paradigm, Franconeri and Simons (2003) showed that moving cues (i.e., looming or translating, but not receding cues) decrease the speed with which participants can detect a target presented immediately afterwards at the same location. In another study, Takeuchi (1997) reported that looming motion captured attention more efficiently, observing that subjects were faster in detecting a looming stimulus within a series of receding ones than the converse condition. Moreover, this facilitation was independent of the number of receding distracters present. Similarly, when participants were required to carry out a reaching task, their movements were more rapid when the targets were preceded by a looming cue, even if this cue did not predict the location of the upcoming target (Franconeri and Simons, 2003; Moher et al., 2014). In addition, von Mühlenen and Lleras (2007) demonstrated that when the effect of abrupt onset, which also captures attention, was canceled out by introducing progressive coherence in a moving random dot pattern, attentional capture occurred only for looming cues. Later, and again using a visual search task, Lin et al. (2009) showed that looming captured attention more efficiently when the stimuli were situated in the peripheral field than when they were placed at the center. Furthermore, they demonstrated that the effect was stronger when the looming movement followed a path that was on a collision course with the viewer, as opposed to trajectories that would not have led to an impact. This latter effect was observed even when the viewer was not aware of the difference between possible collision or not (Lin et al., 2009).

A likely explanation for the heightened attentional capture of looming stimuli may be provided by evolutionary psychology, as the early and rapid detection of looming stimuli appears to be an essential factor for the survival of the individual and, on a larger scale, of the species. Indeed, looming stimuli are highly relevant in that they indicate possible collision with the observer. Stereotypical avoidance behavior, such as head withdrawal and covering of the face with the arms have been observed in monkeys for approaching but not receding or randomly moving stimuli (King and Cowey, 1992). This has also been found in adult humans (King et al., 1992) as well as infants as young as 2–11 weeks of age (Ball and Tronick, 1971). Such defensive behavior and its presence at an early age suggest that this phenomenon is largely automatic and preattentive, and could also operate outside the focus of awareness.

From a general perspective, unconscious processing of visual stimuli has also been observed in patients suffering from cortical blindness. This has been termed “blindsight” and is “a condition in which the sufferer responds to visual stimuli without consciously perceiving them” (Weiskrantz et al., 1974). One patient, DB, who presented right hemianopia, was able to guess correctly if a stimulus was moving in his blind field although he had no conscious experience of “seeing” the stimulus (Weiskrantz, 1986). Subsequently, the neural substrates of motion blindsight were investigated using PET and fMRI imaging techniques, in single cases (Barbur et al., 1993; Zeki and Ffytche, 1998; Goebel et al., 2001; Schoenfeld et al., 2002; Bridge et al., 2010), as well as in group studies (Morland et al., 2004; Barleben et al., 2014; Ajina et al., 2015). The sum of evidence points to the activation of hMT/V5 in response to motion despite the lesions to V1 and in most cases, in the absence of visual awareness.

Less is known about the effects of direction of motion in blindsight, in particular looming motion. In monkeys, looming was investigated after unilateral ablation of the occipital cortex (King and Cowey, 1992). This was experimentally induced with a hoop, the size of a tennis racket that approached the monkeys in their blind, or in their seeing visual half field. Avoidant head movements to the looming stimulus were recorded in two out of three monkeys when presented in their blind (as well as in their seeing) visual half fields. Due to the inability of the primary visual cortex to process visual information, it was hypothesized that this withdrawal response was mediated by the subcortical retinal-superior colliculus (SC) mediated pathway (King and Cowey, 1992).

To the best of our knowledge, no studies have addressed the effect of looming motion in cortical blindness in humans. We therefore examined the response to moving dot patterns in a patient who suffers from complete cortical blindness following bilateral strokes to the posterior cerebral arteries. This patient, TN, has been described elsewhere and was shown to present remarkable residual visual abilities including affective blindsight (Pegna et al., 2005; Burra et al., 2013), as well as relatively preserved navigational abilities (de Gelder et al., 2008) and visuo-motor capacities (Buetti et al., 2013). We investigated TN's BOLD response to dot patterns that were either static, or showed rotational, receding or looming movements. In view of the literature, we expected to observe hMT/V5 activity for motion. Critically, our expectation was that looming motion, in view of its salience, would enhance brain activation, possibly recruiting additional structures that play a part in visual salience.

## Methods

### Case report

TN is a well-studied cortically blind patient that we have recently described in detail (Van den Stock et al., 2014). He suffered two consecutive posterior strokes leaving him cortically blind due to extensive damage to the occipital cortex bilaterally (Pegna et al., 2005). More specifically, the damage to the right hemisphere encompasses the occipital lobe with some sparing of the posterior lingual gyrus and part of the precuneus. The left hemispere damage is larger and extends anteriorly to the middle part of the fusiform gyrus, laterally to the medial inferior temporal gyrus and dorsally up until the superior parietal lobe, leaving the medial ventral part of the inferior occipital gyrus and anterior part of the lingual gyrus intact. The visual perimetry (described in Van den Stock et al., 2014) demonstrated complete blindness. As previously described, the assessment was carried out by presenting small white 1° circles that were either flickering (20 Hz) or static (stimulus luminance 95 cd/m2; duration 300 ms; interstimulus interval 3000 ms). Stimuli were presented on a 17-inch computer screen (background luminance of screen: 2 cd/m2) at 64 equally-spaced positions on a grid that spanned 48° horizontally and 40° vertically in front of the patient. TN had been unable to report the presence of any stimulus.

Clinically, TN continues to behave as a blind person, for example using a cane as a tool to guide himself while walking, locating food on his plate with his fingers, etc…When in presence of his wife or friends, he will rely on them for navigation, for example by placing his hand on their shoulder when walking. It should be pointed out that nothing in his behavior suggests the spontaneous use of vision to guide his actions.

Despite these observations, he sporadically claims to “feel” the occurrence of visual movement. Despite his alleged impressions of visual movement, a careful clinical assessment revealed numerous misses and false positives in his responses. For example, when asked to attend to the possible movement of the examiner's (AP) finger, he would frequently signal its occurrence before any motion had occurred, while on other occasions, he would fail to respond to entirely. Subsequently, in order to determine the extent of actual movement perception that was possible, TN was presented with a series of random dot stimuli consisting of 1000 ms video clips of static or random movement stimuli. Each stimulus consisted of 13 light points placed randomly on the screen that could move randomly at different speeds. TN was instructed to respond if he detected movement or not. He was informed of the presence of the stimulus on the screen by a beep indicating onset and offset. This was followed by a verbal prompt by the examiner.

TN's performance on this task revealed a hit rate of 63%. Out of 192 stimuli presented (96 moving and 96 static stimuli), TN responded correctly to 121 stimuli. With respect to a binomial distribution, this rate yields a Z score of 3.61 with a probability of chance occurrence at *p* < 0.001 (two-tailed). Additionally, a *d*' was computed which yielded a value of 1.43 with a c criterion of 0, demonstrating that the results were not linked to a bias toward a specific response. Consequently, it appears that TN is above chance when detecting moving stimuli although his awareness of motion appears to fluctuate. Of particular importance in the current investigation, TN was unable to describe the type of stimulation that was presented to him in the scanner.

He participated in the present experiment aged 62, i.e., 10 years after the loss of conscious visual perception. He gave his informed consent to participate in this study that was approved by the Ethics Committee of Geneva University Hospital.

### Materials

Videos of dot-fields on a black background were generated to mimic various types of motion, namely: looming, receding, rotation (either clockwise or anticlockwise), and a static condition. Stimuli were back-projected onto a screen of size 40.5 × 24.2 cm with a resolution of 1680 × 1050 pixels, placed behind the participant's head. The participant viewed the screen through a mirror attached to the head coil, with a viewing distance of approximately 75 cm. Dots (diameter 0.18° of visual angle) were presented in a neutral gray (defined by RGB values set to 46.7%) on a black background. Stimuli were generated using Matlab 7.14 (The Mathworks, Natick, MA, USA). Video clips lasted 2 s at 30 frames per second.

**Figure 1:**
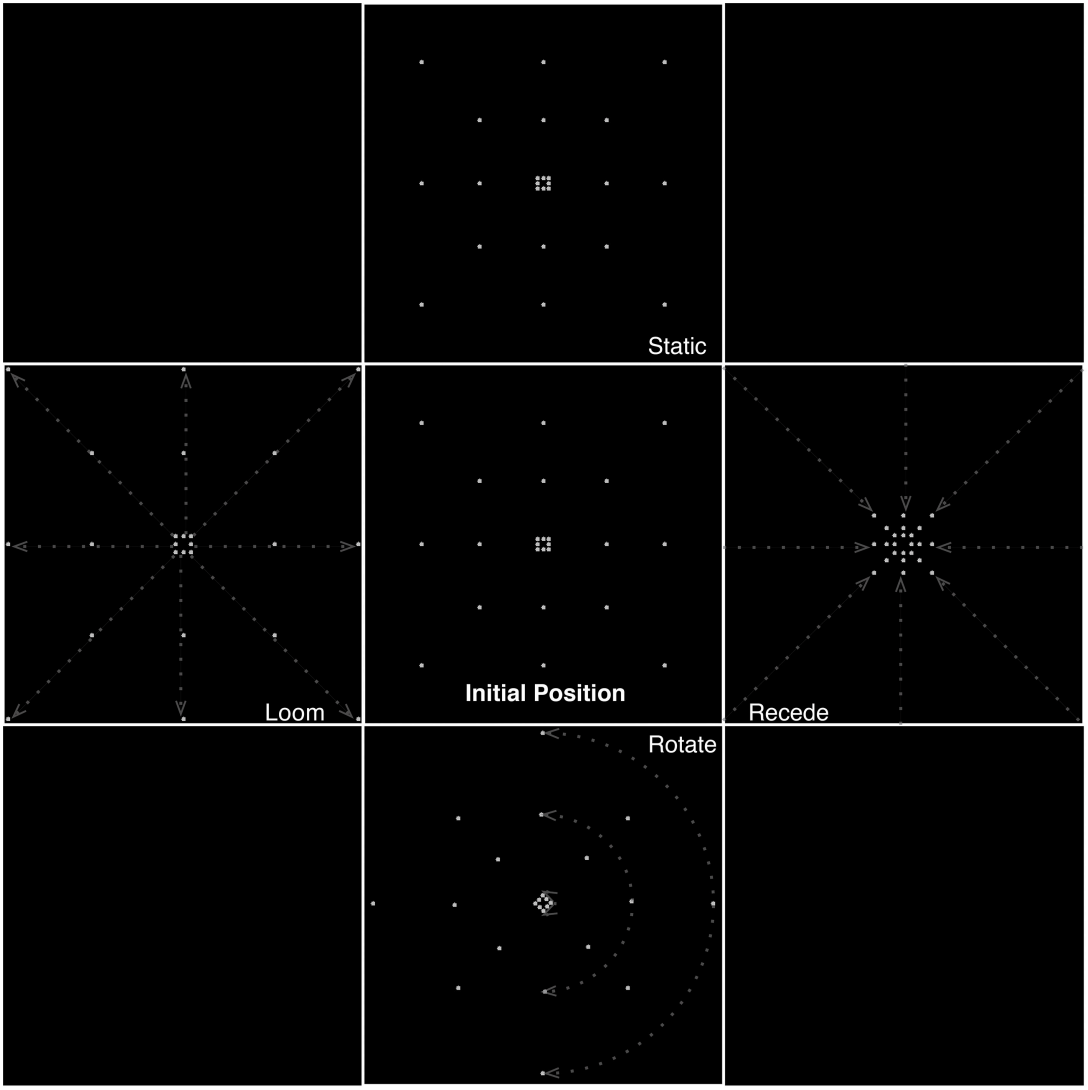
**Different types of motion presented in the experiment, illustrating frames from the static, looming, receding, and rotating conditions**. Dashed lines and arrows indicate illustrative trajectories of dots for each condition. Equiluminance of the stimuli was preserved by maintaining the number of dots at any time in all conditions. In the looming conditions, dots passing the edge of the screen were replaced by dots at the center while for receding stimuli, dots disappearing to the center of the screen were replaced with dots at the edges.

Dots were distributed placed on the perimeter of three imaginary concentric squares. Eight points were placed in this manner, four on the apices and four on the midpoints of each side, such that all stimuli were composed of 24 dots (see Figure 1 for a schematic illustration of the stimuli). Each of the motion conditions was created with four different velocities. Rotating stimuli were presented at rates of 120, 90, 60, and 30°/s. Looming and receding stimuli expanded and contracted by rescaling of the squares defined by the dots at rates (k) of 1.05, 1.1, 1.15, 1.2 per frame (at 30 frames per second at time t, in seconds, the rate of expansion is defined as k^30t^). The rate of change of size therefore described an exponential relationship as a function of proximity to the edge of the screen, increasing closer to the edges. The edges of the smallest square subtended 0.60° of visual angle and those of the largest (extending to almost the corners of the screen) 18.33°.

Equiluminance of the stimuli was preserved by always having the same number of dots present on screen at any time, irrespective of condition (for the looming conditions, dots passing the edge of the screen wrapped around to the center of the screen, and for receding stimuli, dots disappearing to the center of the screen wrapped around to the edges).

A block design was employed in which videos of a given motion type were presented for 16 s (each speed of motion was presented twice in a random sequence, for rotation trials clockwise and anticlockwise stimuli were presented in equal numbers at each speed in a random sequence), followed by a 16 s period of no stimulation (a black screen). Block (i.e., motion condition) sequence was randomized. The fMRI session was divided into four runs, each of which contained two blocks of each motion condition, and the associated null events. Stimulus delivery was controlled using custom routines developed for PsychoPy (Peirce, 2007).

### MRI acquisition, preprocessing, and analysis

Anatomical and functional MR images were acquired in Maastricht University, with a 3T whole-body scanner (Magnetom Trio, Siemens, Erlangen, Germany) equipped with a 20-element head-neck coil.

T2^*^-weighted functional images were acquired using a gradient echo EPI sequence, with 2 × 2 × 2 mm^3^ resolution, covering the whole brain (75 slices without gaps, *TR* = 2000 ms, *TE* = 31 ms, flip angle = 62, multiband acceleration factor = 3, FOV = 172 × 172, matrix size = 96 × 96). Sixteen scans were acquired per condition per session, for a total of 64 per condition over the experiment.

In addition, a T1-weighted anatomical image (1 × 1 × 1 mm^3^ isotropic) was acquired with using an MPRAGE sequence (*TR* = 2250 ms, *TE* = 2.17 ms).

Stimuli were back-projected onto a screen placed behind the participant's head. The participant viewed the screen through a mirror attached to the head coil, with a viewing distance of approximately 75 cm. The patient was asked to keep his eyes open and oriented straight ahead during the experimental presentation. His direction of gaze was monitored with an eye tracker camera, although it was not possible to obtain calibration values due to his inability to see the calibration points.

Preprocessing and analysis of the data was carried out in SPM8 (http://www.fil.ion.ucl.ac.uk/spm/). Data preprocessing proceeded as follows:

1. Rigid realignment of each EPI volume to the first in the session, using a two-pass procedure in which the images are realigned to the mean image created after a first-pass realignment,
2. Coregistration of the MPRAGE image to the mean EPI image,
3. Spatial smoothing using a Gaussian kernel of 8 mm at full-width half-maximum height.

In order to be able to compare the patient's neuroanatomy with that of existing atlases, we normalized the T1 anatomical image to the standard MNI template included in SPM8, using the “unified segment” procedure (Ashburner and Friston, 2005), which is relatively robust in the face of lesions (Crinion et al., 2007). The normalization parameters generated by this process were subsequently applied to the contrast images produced by the functional analysis. Activation co-ordinates are reported in MNI space and region identification was assisted by use of the AAL (automated anatomical labeling, Tzourio-Mazoyer et al., 2002) and Brodmann area templates, both supplied with MRIcroN (Rorden, 2011), more precise denominations were obtained using the Anatomy toolbox (version 20, retrieved from http://www.fz-juelich.de/inm/inm-1/EN/Forschung/_docs/SPMAnatomyToolbox/SPMAnatomyToolbox_node.html Eickhoff et al., 2007).

The preprocessed EPI images were submitted to a GLM analysis, in which each scan was coded for a condition (looming, receding, rotating, static) and null events were left unmodeled (after Josephs and Henson, 1999). Each of the four sessions was modeled separately and the effect of session was coded separately. Each event was modeled using the canonical haemodynamic response function in SPM8. Six parameters were appended in each of the sessions to code for the effects of movement (x, y, and z translations and x, y, and z rotations derived from the rigid realignment step of the pre-processing). A high-pass filter (cutoff 128 s) and AR1 correction for serial autocorrelation were applied. The parameter estimates of the GLM were then compared to obtain contrast images revealing the differences in BOLD response between the various conditions.

### Control participants

A control group, composed of eight right-handed male participants (mean age: 58 years, range: 55–62) with normal or corrected-to-normal vision and no history of head injury was scanned using the same experimental paradigm. Control participants were scanned at Geneva University Hospitals, on a 3T Siemens Prisma, with a 20-channel head-and-neck coil. Screen-size and resolution for stimulus presentation were as for TN. Since, for operational reasons, these participants could not be scanned on the same scanner as TN, only qualitative comparisons of these data will be presented.

The control participants' data was analyzed at the first, fixed-effects level as TN's data. In order to obtain estimates of reliable activity over the group, contrast estimates from each of the participants were subsequently analyzed at the second, random-effects level. Second-level analysis comprised a repeated-measures ANOVA, in which the first-level condition vs. baseline images for each participant were entered into a design matrix to test for between-condition differences. In order to account for between-participant variability, “subject” was included as a factor in the analysis. Analysis was carried out in SPM8, following the methods described by Henson and Penny (2005).

## Results

Since the primary focus of this paper is on the detection of looming stimuli by the brain's subcortical pathways, analyses will focus on the areas selectively activated by these stimuli, and particularly in contrast to those engaged by receding stimuli. The effect of speed of movement on BOLD response in each of the different movement conditions was tested and no significant effects were found. As a result, this condition was collapsed.

For completeness, results are presented at a relatively liberal threshold of uncorrected *p* < 0.0001 with a cluster-extent threshold (K) empirically determined (using a modified script retrieved from: http://www-personal.umich.edu/~nichols/JohnsGems2.html) for each contrast that is presented using a Newton Raphson search procedure to yield clusters significant at *p*_(FWE)_ < 0.05 at the cluster level. Results that are discussed are significant at *p*_(FWE)_ < 0.05 either at the voxel level or the cluster level (based on the cluster extents at uncorrected *p* < 0.0001).

### Looming vs. receding

**Figure 2:**
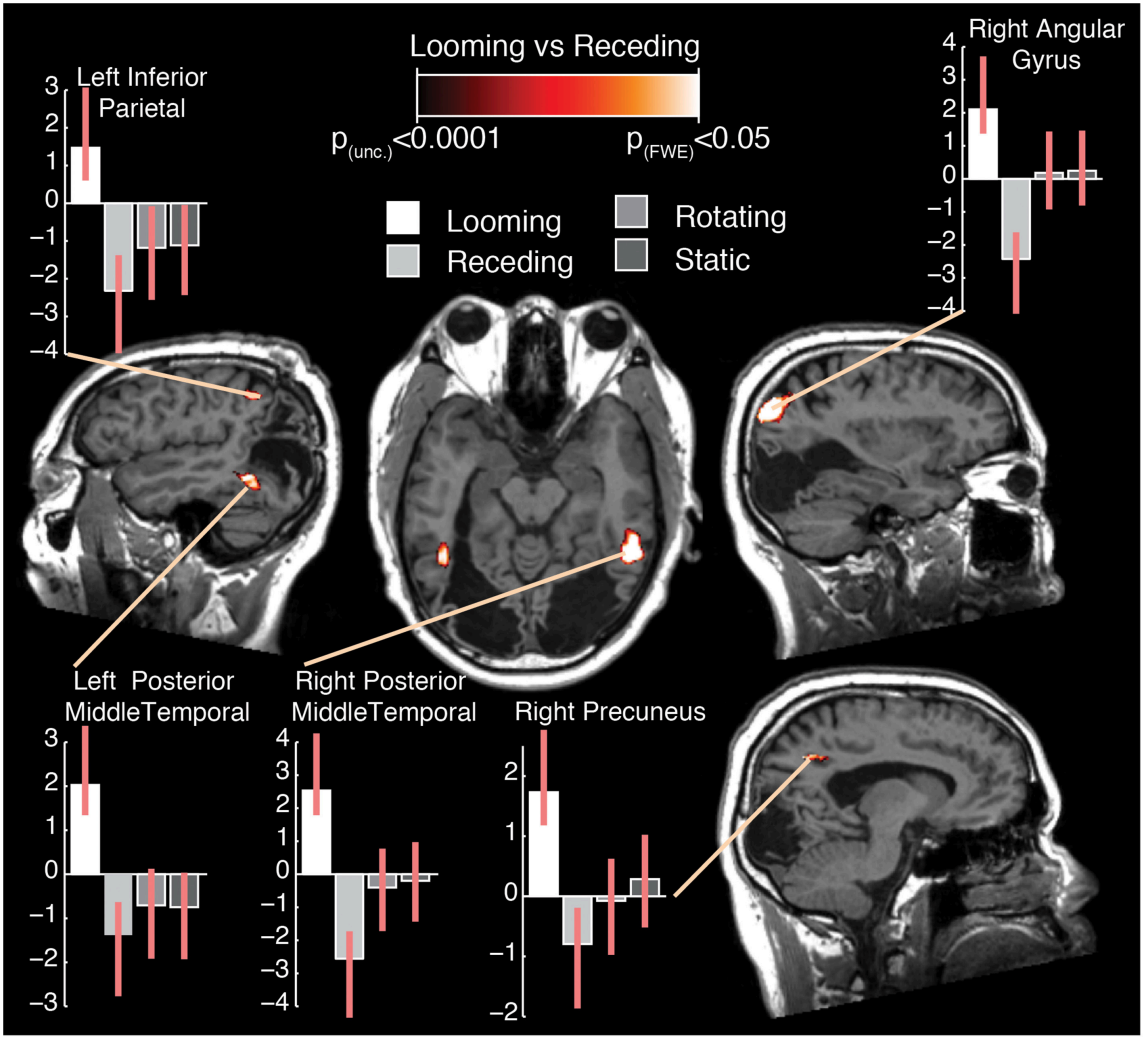
**Selected slices of the T1 anatomical scan, upon which are projected the clusters of significant BOLD response difference for the contrast Looming—Receding, thresholded at ***p*** (uncorrected) < 0.0001, with a cluster extent threshold (***k***) of 75 voxels**. This value of *k* was empirically determined to be the level for which clusters composed of voxels at least meeting uncorrected *p* < 0.0001 would reach familywise-error corrected significance at the cluster level. No co-ordinates are given here as the images are presented in the patient's native space. For comparison with previously published data and atlases, the co-ordinates of the activation differences are presented normalized to MNI-space in Table 1. Graphs indicate the relative activation relative to baseline for each movement condition, error bars indicate 95% confidence intervals.

The subtraction of looming and receding stimuli revealed a difference in BOLD response in a number of areas (see Figure 2; Table 1). A significant increase in activation was found in bilateral posterior middle temporal regions. This cluster was extensive in both hemispheres, covering a large region of the posterior middle temporal gyrus through the inferior temporal cortex. A further substantial cluster of difference in BOLD response was found in the right parietal lobe, centered about the angular gyrus. A cluster of slightly right-lateralised activation difference was observed in the precuneus and the middle cingulate cortex. Finally, a difference in activation for looming vs. receding stimuli was found in the left inferior parietal lobule.

**Table 1:** **Summary of results for the contrast looming—receding in Patient TN**.

**Region name [brodmann area]**	**Hem**	**MNI co-ordinates**			***T*-Value**	***Z*-Score**	**Cluster size**
		***x***	***y***	***z***			
**Posterior inferior temporal gyrus [BA20]**	**R**	**54**	−**50**	−**14**	**6.07**	5.99^*^	**520**^†^
Posterior middle temporal gyrus [BA20]	R	50	−36	−10	4.25	4.22	
Cerebellum crus I [BA37]	R	52	−50	−30	4.02	3.99	
**Angular gyrus [BA7]**	**R**	**38**	−**70**	**40**	**5.72**	5.65^*^	**595**^†^
Angular gyrus [BA7]	R	36	−62	42	4.83	4.79^*^	
**Posterior middle temporal gyrus [BA37]**	**L**	−**46**	−**52**	−**2**	**4.97**	4.92^*^	**303**^†^
Occipitotemporal area [BA37]	L	−42	−60	−10	4.76	4.72^*^	
Inferior occipital cortex [BA19]	L	−38	−76	−6	4.18	4.15	
Posterior superior temporal sulcus [BA21]	L	−44	−46	10	4.15	4.12	
**Precuneus**	**R**	**16**	−**54**	**42**	**4.7**	4.66^*^	**278**^†^
Middle cingulate gyrus [BA23]	R	2	−40	36	4.66	4.62^*^	
Precuneus	R	16	−44	40	4.2	4.17	
**Inferior parietal lobule [BA40]**	**L**	−**44**	−**46**	**48**	**4.54**	**4.51**	**96**^†^

*Only clusters with peak voxels reaching p_(FWE)_ < 0.05, or clusters attaining cluster-level corrected p_(FWE)_ < 0.05 (empirically determined to be those containing >75 voxels using a Newton-Raphson search procedure) are reported in this table. Sub-peaks of clusters a minimum of 8 mm apart are reported with a threshold of uncorrected p < 0.0001. Co-ordinates are in mm in MNI space. Bold rows indicate the peak voxel of each cluster. Hem denotes the hemisphere of the activation*.

* *indicate voxels significant at a voxelwise FWE-corrected p < 0.05*.

† *indicate clusters significant at p < 0.05 corrected for multiple comparisons at the cluster level*.

It can be seen from the plots of relative activation levels presented in Figure 2 that the pattern of responses to the various categories of motion in these regions is broadly similar. In essence, there is an elevated response to looming stimuli, a decrease in BOLD contrast in response to receding stimuli, and a minimal response to rotating or static stimuli. This pattern of responses suggests that the residual visual processing pathways in TN are specifically sensitive to the relatively more-salient looming stimuli. While it can be difficult to interpret decreases in BOLD response meaningfully, they have been demonstrated to index neural deactivation in early visual cortex (Pasley et al., 2007). If this is indeed the case, then it suggests that the areas showing negative BOLD responses here are sensitive to the direction of movement of dot fields, specifically as a function of their apparent looming.

Surprisingly and in sharp contradistinction with TN, control participants did not manifest any significant increase in BOLD response to looming over receding stimuli.

In order to determine whether any differential activation was reliably present for movement and therefore if a differential BOLD response could be observed in comparison with baseline, we examined the main-effect of motion in both TN and the control group. This result is described in the next section.

### Main-effect of movement

**Figure 3:**
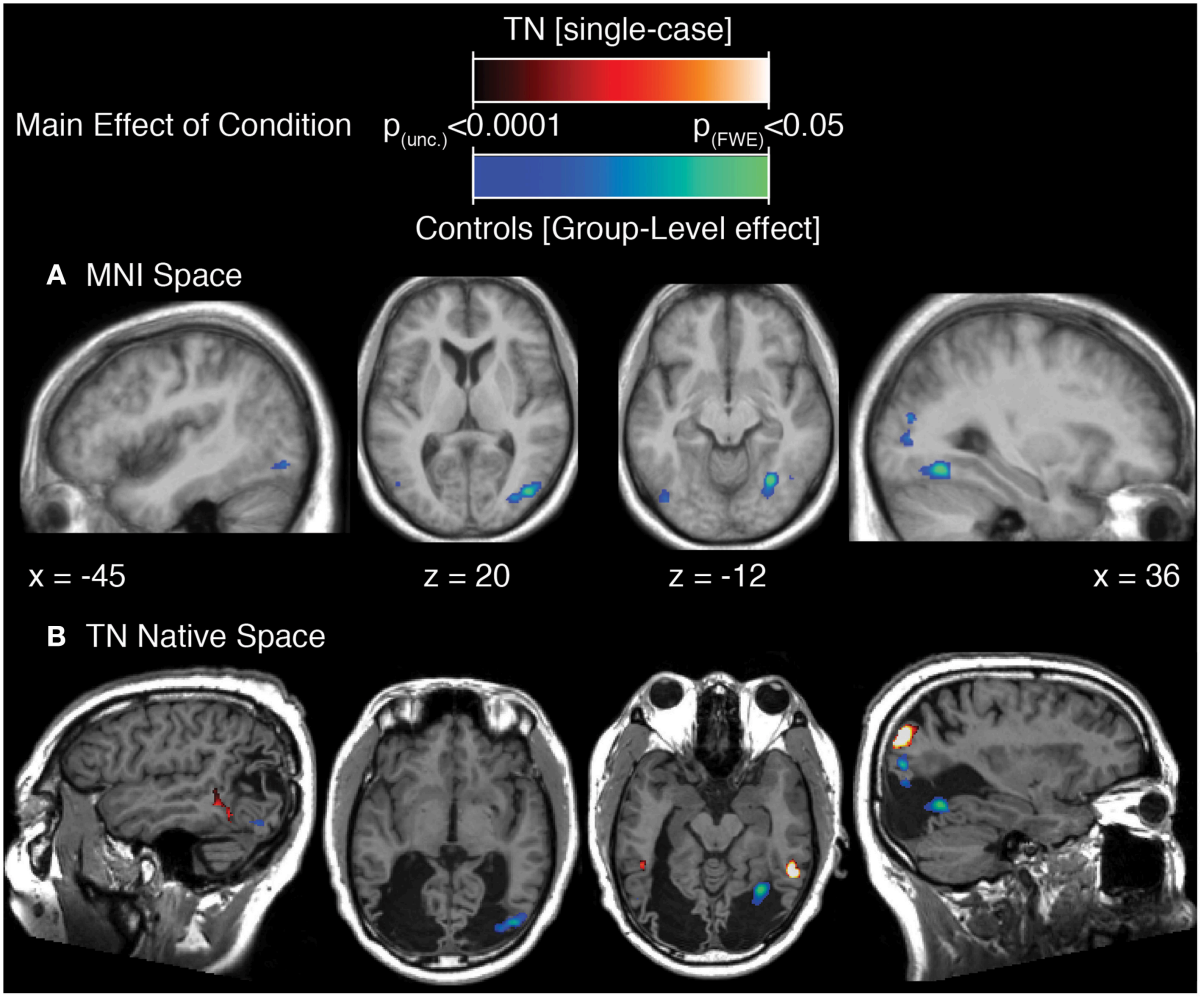
**(A)** Main effect of condition in the control participants (repeated-measures ANOVA, four levels: Loom, Recede, Rotate, Static), showing significant differences in BOLD response thresholded at *p* (uncorrected) < 0.0001, with a cluster extent threshold (*k*) of 70 voxels. This value of *k* was empirically determined to be the level for which clusters composed of voxels at least meeting uncorrected *p* < 0.0001 would reach familywise-error corrected significance at the cluster level. Data are projected onto the average T1 structural image of the control participants, normalized to MNI space. Co-ordiantes indicate plane of section of each slice. **(B)** The main-effect of condition in the control participants as in **(A)**, but projected into TN's native space, displayed upon TN's T1 structural scan, and presented alongside the map of significant main effect of condition for TN (thresholded as for controls, k was determined to be 77).

The results of this analysis is illustrated in Figure 3A; Table 2A for the control group and in Figure 3B; Table 2B for TN. In controls, significant stimulus-induced differences in BOLD response are seen in extrastriate visual regions, corresponding to the fusiform gyrus on the right and to MT/V5 bilaterally (Figure 3A). In order to compare the locus of differential motion-sensitivity in the controls and TN, the map of the control group's significant activation was warped to TN's native space (Figure 3B). A side-by-side comparison of the controls' and patient's activation patterns indicates that the locus of TN's possibly-equivalent posterior middle temporal response is anterior to that of the controls, and the response observed in the inferior parietal lobe (IPL) is situated dorsal to that of the controls. It is also worth noting that some portions of the control group's response fall inside the region of TN's lesion.

**Table 2A:** **Summary of results for the main effect of condition in the control group**.

**Region name [Brodmann area]**	**Hem**	**MNI co-ordinates**			***F*-Value**	***Z*-Score**	**Cluster size**
		***x***	***y***	***z***			
**Fusiform gyrus [BA 19], area FG1**	**R**	**30**	−**62**	−**10**	**25.84**	4.99^*^	**246**^†^
Fusiform gyrus [BA18], V4	R	26	−74	−6	14.58	4.07	
Inferior occipital [BA37], area FG2	R	44	−62	−12	12.72	3.85	
**Middle occipital gyrus [BA19]**	**R**	**34**	−**78**	**18**	**24.51**	4.91^*^	**479**^†^
Middle occipital gyrus [BA19]	R	44	−76	6	24.4	4.9^*^	
Posterior middle temporal [BA39],	R	46	−72	20	22.56	4.77	
Middle occipital [BA18]	R	34	−82	6	18.45	4.45	
Inferior temporal [BA19], V5/MT	R	44	−70	−2	12.4	3.81	
Middle occipital [BA18]	R	26	−82	10	12.29	3.79	
**Inferior occipital [BA19], area FG2**	**L**	−**44**	−**78**	−**10**	**17**	**4.32**	**80**^†^
Middle occipital [BA37], V5/MT	L	−48	−72	2	13.81	3.98	

Only clusters with peak voxels reaching p_(FWE)_ < 0.05, or clusters attaining cluster-level corrected p_(FWE)_ < 0.05 (empirically determined to be those containing >70 voxels using a Newton-Raphson search procedure) are reported in this table. Sub-peaks of clusters a minimum of 8 mm apart are reported with a threshold of uncorrected p < 0.0001. Co-ordinates are in mm in MNI space. Bold rows indicate the peak voxel of each cluster. Hem denotes the hemisphere of the activation

* *indicate voxels significant at a voxelwise FWE-corrected p < 0.05*.

† *indicate clusters significant at p < 0.05 corrected for multiple comparisons at the cluster level*.

**Table 2B:** **Summary of results for the main effect of condition in Patient TN**.

**Region name [Brodmann Area]**	**Hem**	**MNI co-ordinates**			***F*-Value**	***Z*-Score**	**Cluster size**
		***x***	***y***	***z***			
**Inferior temporal gyrus [BA20]**	**R**	**54**	−**50**	−**14**	**13.55**	5.56^*^	**262**^†^
**Middle occipital gyrus [BA19], area PGp**	**R**	**38**	−**72**	**38**	**11.99**	5.17^*^	**321**^†^
Angular gyrus [BA7]	R	34	−64	44	8.98	4.33	
**Middle temporal gyrus [BA37]**	**L**	−**48**	−**52**	**0**	**9.89**	**4.59**	**149**^†^
Inferior occipital gyrus [BA37], area FG2	L	−42	−60	−10	9.46	4.47	
Middle temporal gyrus [BA21]	L	−44	−48	10	8.35	4.13	

*Only clusters with peak voxels reaching p_(FWE)_ < 0.05, or clusters attaining cluster-level corrected p_(FWE)_ < 0.05 (empirically determined to be those containing >77 voxels using a Newton-Raphson search procedure) are reported in this table. Sub-peaks of clusters a minimum of 8 mm apart are reported with a threshold of uncorrected p < 0.0001. Co-ordinates are in mm in MNI space. Bold rows indicate the peak voxel of each cluster. Hem denotes the hemisphere of the activation*.

* *indicate voxels significant at a voxelwise FWE-corrected p < 0.05*.

† *indicate clusters significant at p < 0.05 corrected for multiple comparisons at the cluster level*.

## Discussion

This study investigated the cerebral response to moving (looming) stimuli in a patient (TN) who became cortically blind after having suffered a bilateral loss of his primary visual cortex. In addition, an age- and gender-matched control group was scanned for comparison purposes. The results in TN showed that looming activated a network that included the posterior middle temporal lobes bilaterally, the left inferior parietal region, the right angular gyrus and the right precuneus. This occurred despite the fact that the patient could not detect the direction of motion of the stimuli or even their presence. Surprisingly, data obtained in the control group failed to produce any significant activation for looming, although motion *per se* produced a significant effect in areas MT/V5.

The importance of the motion selective region MT/V5 in humans was identified after a patient with bilateral damage to these areas was described. The patient was subsequently impaired in motion processing, suffering from what is known as akinetopsia (Zihl et al., 1983, 1991). In healthy controls, the first brain imaging techniques addressing the question of cerebral specialization for visual processing found activation in the same regions in response to moving stimuli (Zeki et al., 1991), confirming the specificity of this area for motion processing. The activations found in our control group for motion are therefore entirely consistent with the literature and add to the large corpus of existing reports showing an increased response in area MT/V5 for movement.

By contrast, data from TN was slightly more surprising. Indeed, activation for motion in TN was not comparable to controls. The superimposition of the control group's regions of activation onto TN's brain revealed a certain amount of overlap of these clusters with the damaged areas. In TN, regions of significant activation for motion were in fact situated in the left middle temporal gyrus, the right inferior temporal gyrus and the angular gyrus. Furthermore, looming yielded several distinct areas of activation, including two clusters in the posterior middle temporal regions, one of them (on the left) coinciding with the area activated for movement *per se*.

The activation of these middle temporal areas in TN were rather unexpected. Indeed, we had anticipated the activation of more posterior regions compatible with MT/V5. However, even allowing for the possibility of errors occurring in the normalization and warping procedures, the localization of the middle temporal clusters appears too posterior and therefore do not appear to be compatible with known hMT+ regions in the healthy brain (Watson et al., 1993; Dupont et al., 1994; Goebel et al., 1998; Sunaert et al., 1999). Nevertheless, the fact that the (left) middle temporal region in TN responds to the movement conditions, and that TN's lesion extends toward and partly overlaps with MT/V5 locations in our controls, suggests that cortical plasticity may have led to changes in the regions surrounding the lesion. As a result, more anterior areas may have become involved in motion processing through neural projections from subcortical structures and could thus have partly taken over the functions normally subserved by MT/V5. Further investigation would however be necessary to confirm this suggestion. As noted above, investigations of residual visual motion processing in cases of hemianopia have revealed activation in regions compatible with hMT/V5. Zeki and Ffytche (1998) studied patient GY's response to moving stimuli using PET, and established a response in what appeared to be area hMT/V5. This was subsequently confirmed with fMRI in patient GY, as well as in two other patients with hemianopia (Goebel et al., 2001; Schoenfeld et al., 2002). More recently, Bridge et al. (2010) studied a patient with bilateral partial loss of the striate cortex and found hMT/V5 activation bilaterally, but none in V1, in response to random dot kinematograms. Furthermore, the authors observed intact tracts between LGN and hMT/V5, while those between LGN and V1 showed evidence of degeneration. This finding corroborates the hypothesis that hMT/V5 may receive information through a direct pathway from the LGN. Indeed, one of the two dominant views has suggested that neurons from the LGN may project directly to extrastriate visual cortex, possibly from intralaminar k-cells, allowing information to be processed independently of V1 (Cowey and Stoerig, 1989; Schmid et al., 2010). Alternatively, a second influential view suggests that a subcortical pathway to the SC and pulvinar, and from there to the extrastriate region may be the most likely route allowing V1-independent processing to take place (Rodman et al., 1990; Kato et al., 2011). In line with this hypothesis, in a very recent fMRI study (Barleben et al., 2014), BOLD response (and diffusion tensor imaging –DTI–) was measured in eight hemianopics during stimulation by moving bars. Six out of eight patients showed an hMT response to motion, and both lesional as well as DTI results pointed to the pulvinar as a likely component of the pathway, echoing previous findings in healthy controls (Lanyon et al., 2009).

More recently, another hypothesis has suggested that blindsight may be linked to the transfer of visual information between healthy and lesioned hemispheres (Leh et al., 2006; Bridge et al., 2008; Silvanto et al., 2009). In view of the bilateral lesions in TN, this interpretation cannot be upheld as neither hemisphere is able to process visual information through V1.

Our current findings do not allow us to determine whether the geniculo-extrastriate or colliculo-pulvinar routes are involved in conveying information to the cortical regions activated in TN's brain. Nevertheless, activation was observed in the middle temporal regions (bilaterally for looming and on the left for motion) demonstrating the recruitment of typically non-visual cortex for movement processing and suggesting that cortical reorganization may have led more anterior regions to perform some of the functions usually performed by hMT+/V5, and more importantly, showing a heightened response to radially approaching movement.

The increased sensitivity of modality-specific cortical regions for looming has been observed on different occasions. For example, in a study addressing looming from a multimodal perspective, Tyll et al. (2013) compared auditory and visual looming and receding stimuli in healthy subjects. Looming stimuli enhanced the fMRI signal in both the visual and auditory cortices. Additionally, multisensory audiovisual looming stimuli enhanced the STS region and the visual cortex in a superadditive manner. This cortical coupling effect was explained by a psychophysiological interaction analysis that showed increased connectivity between the seeded STS and the visual cortices during looming compared to receding stimuli (Tyll et al., 2013). Thus, cortical feedback connections were hypothesized to be at play during the processing of these salient stimuli (Tyll et al., 2013).

In another brain imaging study with healthy controls, the existence of a collicular route for processing of looming was evidenced. Billington et al. (2011) compared brain activation in response to a moving ball made of point lights under conditions of looming, receding and random motion. In addition to a large cortical network, looming enhanced the response of the SC, as well as the medial pulvinar, corroborating the view that a collicular pathway might convey information to cortical regions for unconscious processing of motion when V1 is damaged.

The evidence for an enhanced activation of modality-specific cortical regions in response to looming is in line with TN's heightened cortical response. The preference of the sensory system for looming stimuli shows that an organism is biased toward stimuli reflecting possible collisions and thus potential threat. In point of fact, such enhanced processing for threatening stimuli has received much attention in the field of affective science, and findings have revealed that stimuli such as threatening facial expressions enhance stimulus-specific visual cortical areas and can in fact do so both when stimuli are consciously detected or not (Vuilleumier et al., 2004; Rotshtein et al., 2010; Van den Stock et al., 2013, 2014).

Little is known about whether looming may be processed in the absence of awareness. One study addressed this using a binocular rivalry task. In binocular rivalry, the viewer can usually only report the stimulus in one of the two eyes, while the stimulus in the other is not consciously detected. Although the conscious percept shifts from one eye to the other in equal proportions, a more salient stimulus tends to be consciously perceived more often, catching the viewer's awareness. Following this line of reasoning, Parker and Alais (2007) presented receding stimuli to one eye and approaching stimuli to the other. They found that participants reported the looming stimuli for longer durations, demonstrating that they attracted attention and broke into awareness more easily. Consequently, looming must be processed prior to awareness. Similar conclusions have been reached with patients suffering from brain damage. When patients with right parietal lobe lesions and visual extinction are presented with stimuli in the left and right spatial field simultaneously, they cannot report the stimulus in their extinguished (left) field. Dent and Humphreys (2011) showed that during bilateral stimulus presentations, looming overcame extinction. Patients reported the presence of the expanding, but not the contracting stimuli presented on the left, again showing that looming must be processed pre-attentively and prior to awareness. Rather unfortunately, we were unable to test TN behaviorally with looming stimuli. Nevertheless, on the basis of the fMRI results obtained in our study, we hypothesize that the saliency of the looming stimuli and their behavioral relevance form the basis for the heightened response, which must occur through a parallel non-striate route, allowing them to be processed without awareness and without V1.

Consistent with the idea of a saliency-driven response, our findings in TN additionally pointed to an involvement of the IPL, an area that also appears to be strongly linked with both saliency and multisensory processing. Data from the IPL is derived in large part from studies in the ventral inferior parietal area (VIP) of the monkey, which is located in the depth of the intraparietal sulcus (Bremmer et al., 2001; Guipponi et al., 2013). The VIP has been identified as a visual motion processing area, essentially processing visual stimuli, but also possessing neurons that respond to auditory and somatosensory, as well as multimodal stimulation (Hyvärinen, 1981; Duhamel et al., 1998; Bremmer et al., 2001; Guipponi et al., 2013).

Motion selective cells recorded in area VIP appear to be sensitive to the direction of the light points moving across the visual field (expanding/approaching vs. contracting/receding or other movement), and of particular relevance in our study, to stimuli representing real or apparent self-motion (Colby et al., 1993; Bremmer et al., 2002). Even more significant for our purpose, neurons in monkeys' area VIP have been found to be sensitive to the position of the movement relative to the observer, and to respond differentially according to whether the apparent location of the movement is situated in near or far extrapersonal space. Neuronal responses were found to be strongest for close, head-centered, approaching stimuli, in comparison to the responses to stimulation in the more distant extrapersonal space (Colby et al., 1993; Bremmer et al., 2013). The fact that approaching visual stimuli in the near, head-centered, personal space trigger neuronal responses, highlights the role of this area in guiding head movements in space, as well as in assessing the salience of moving stimuli and in preparing for avoidance reactions (Colby et al., 1993; Bremmer et al., 2013). This interpretation is supported by evidence showing that monkeys display defensive behaviors such as protecting the face with their arms following VIP electrical stimulation (Cooke and Graziano, 2004; Graziano and Cooke, 2006). Previous behavioral observations in TN have demonstrated his surprising unconscious ability to navigate through a trajectory while avoiding obstacles (de Gelder et al., 2008). The parietal brain areas activated by the looming stimuli may well provide the cerebral substrate that allowed his navigational abilities to occur.

It has been proposed that a high level, saliency-based motion mechanism may be opposed to low level, luminance-based processes (Cavanagh, 1992) and would be neuroanatomically segregated into saliency-based IPL and low level MT/IPS/STS processing (Claeys et al., 2003). Claeys et al. (2003) investigated visual saliency by creating an oddball paradigm in which stimulus features were manipulated, e.g., by increasing the amount of green contribution to a red and green grating stimulus. Saliency was found to produce IPL activation in support of their hypothesis. In monkeys, single cell recordings from the IPL showed that saliency-based visual information processing depended both on the sudden onset of a stimulus and the behavioral relevance of the stimulus (Gottlieb et al., 1998). Furthermore, the authors discuss that cells in the primary visual cortex always fire when the appropriately-oriented stimulus is present in the receptive field independently of salience, whereas in the IPL, information that is not behaviorally relevant seems to be filtered out before or within the IPL. Our current findings would seem to argue against such an interpretation. Indeed, patient TN lacks both visual cortices yet IPL activation is present for our salient, looming stimuli. This suggests that the saliency of a moving stimulus can be processed in the IPL, independently of prior processing in the primary visual cortex.

Interestingly, a hemispheric specialization dissociation concerning saliency based attentional processes has been put forward (Mevorach et al., 2006b). Whereas, TMS over the right IPL was shown to disrupt attentional engagement toward a salient stimulus while participants made judgments in a compound letter task, left IPL caused participants to be unable to disengage from a salient stimulus. The same authors then replicated their finding with patients suffering from left IPL lesions and found that they were unable to disengage from the salient stimulus in a similar task (Mevorach et al., 2006a). Accordingly, we speculate that our data showing left IPL activation to looming stimuli could reflect TN's attentional capture and inability to disengage from the salient looming stimulus, even though V1 input is lacking.

The presence of middle cingulate activity for looming motion in TN is notable. Several studies in healthy controls have found activity in the posterior cingulate for moving stimuli, in particular when coherent motion was represented (Sunaert et al., 1999; Braddick et al., 2001; Dieterich et al., 2003; Orban et al., 2003; Stebbins et al., 2004; Stiers et al., 2006). Antal et al. (2008) further observed that this region was more strongly activated for what they termed complex coherent motion (i.e., radial and rotational) than simple coherent motion such as horizontal or vertical motion of point lights. In our findings, the posterior cingulate failed to show up in the healthy controls or in TN during motion *per se*, but in TN, looming produced a greater response in this area than receding and rotational motion. The explanation for this particular pattern of response is unclear, however the posterior cingulate has been implicated as part of a network involved in orientation (Berthoz, 1997; Kataoka et al., 2006; Vogt et al., 2006) and evidence from neuropsychology has shown that damage to the posterior cingulate cortex gives rise to difficulties in spatial navigation and heading (Maguire, 2001). This deficit may result from an inability to integrate egocentric visual stimulation and spatial representations for navigational purposes (Vogt et al., 1992) suggesting that this region constitutes part of a larger neural network playing a role in spatial abilities, in which visual cues indicating the position of the stimulus with respect to the body are essential.

In conclusion, our findings revealed that, despite the absence of a functional primary visual cortex, looming motion activated a neural network that included bilateral clusters in the middle temporal lobes anterior to hMT+/V5 areas in the healthy brain that may have developed through cortical plasticity to respond to motion. In addition, cortical regions that generally participate in the processing of visual saliency (IPL) and visual guidance in space (IPL and cingulate) were found to be active. The fact that looming may continue to be processed without V1 may be due to its high level of salience and the importance of this direction of motion as a signal of imminent collision or threat.

### Conflict of interest statement

The Review Editor Holly Bridge declares that, despite being affiliated to the same institution as author Marco Tamietto, the review process was handled objectively and no conflict of interest exists. The authors declare that the research was conducted in the absence of any commercial or financial relationships that could be construed as a potential conflict of interest.
